# Protocol of an ongoing randomized controlled trial of care management for comorbid depression and hypertension: the Chinese Older Adult Collaborations in Health (COACH) study

**DOI:** 10.1186/s12877-018-0808-1

**Published:** 2018-05-29

**Authors:** Shulin Chen, Yeates Conwell, Jiang Xue, Lydia W. Li, Wan Tang, Hillary R. Bogner, Hengjin Dong

**Affiliations:** 1 Department of Psychology, Zhejiang University, Hangzhou, China; 2 Department of Psychiatry, University of Rochester Medical Center, 300 Crittenden Boulevard, Rochester, NY 14642 USA; 3 School of Social Work, University of Michigan, Ann Arbor, USA; 4 Department of Global Biostatistics and Data Science, Tulane University, New Orleans, USA; 5 Department of Family Medicine, University of Pennsylvania, Philadelphia, USA; 6 Faculty of Public Health Research, Zhejiang University, Hangzhou, China

**Keywords:** Depression, Hypertension, Older adults, Rural China, Collaborative care

## Abstract

**Background:**

Depression and hypertension are common, costly, and destructive conditions among the rapidly aging population of China. The two disorders commonly coexist and are poorly recognized and inadequately treated, especially in rural areas.

**Methods:**

The Chinese Older Adult Collaborations in Health (COACH) Study is a cluster randomized controlled trial (RCT) designed to test the hypotheses that the COACH intervention, designed to manage comorbid depression and hypertension in older adult, rural Chinese primary care patients, will result in better treatment adherence and greater improvement in depressive symptoms and blood pressure control, and better quality of life, than enhanced Care-as-Usual (eCAU). Based on chronic disease management and collaborative care principles, the COACH model integrates the care provided by the older person’s primary care provider (PCP) with that delivered by an Aging Worker (AW) from the village’s Aging Association, supervised by a psychiatrist consultant. One hundred sixty villages, each of which is served by one PCP, will be randomly selected from two counties in Zhejiang Province and assigned to deliver eCAU or the COACH intervention. Approximately 2400 older adult residents from the selected villages who have both clinically significant depressive symptoms and a diagnosis of hypertension will be recruited into the study, randomized by the villages in which they live and receive primary care. After giving informed consent, they will undergo a baseline research evaluation; receive treatment for 12 months with the approach to which their village was assigned; and be re-evaluated at 3, 6, 9, and 12 months after entry. Depression and HTN control are the primary outcomes. Treatment received, health care utilization, and cost data will be obtained from the subjects’ electronic medical records (EMR) and used to assess adherence to care recommendations and, in a preliminary manner, to establish cost and cost effectiveness of the intervention.

**Discussion:**

The COACH intervention is designed to serve as a model for primary care-based management of common mental disorders that occur in tandem with common chronic conditions of later life. It leverages existing resources in rural settings, integrates social interventions with the medical model, and is consistent with the cultural context of rural life.

**Trial registration:**

ClinicalTrials.gov ID: NCT01938963; First posted: September 10, 2013.

## Background

China’s population of older adults is growing rapidly. Estimates are that by 2015 there will be over 140 million people in China over age 65 years—equivalent to 44% of the total U.S. population (U.S. Census). The prevalence of affective illness among older adults is significant. Almost 6% of community-dwelling Chinese over age 60 have a depressive disorder [1, 2]. Among those with chronic medical conditions, the prevalence of depression is higher, about 13% [3]. Depression is associated with functional impairment, greater use and higher costs of healthcare, and increased risks of suicide and all-cause mortality [4].

Hypertension (HTN) is one of the most common chronic conditions in China, estimated to have affected 57% of rural village residents over age 60 [5]. HTN is a major cause of strokes and ischemic heart disease [6], which is the only disorder to top depression in the WHO’s list of greatest expected causes of global illness burden by 2020 [7].

Depression and HTN commonly coexist [3, 8, 9]. A study in Shanghai shows that over 78% of medical inpatients with HTN had clinically significant depressive symptoms [10]. The combination of HTN and depression complicates treatment; for example, depression may affect patients’ hypertension medication adherence. However, there is low recognition of the need to treat depression in China [10, 11], and mental health care in general is difficult to access, especially in rural areas. Primary care providers (PCP) in rural villages of China are charged with managing their patients’ chronic conditions, including HTN, but they receive little or no training in diagnosis or treatment of mental illnesses and are not permitted to prescribe antidepressants.

Approaches to management of depression [12–14] and comorbid medical conditions [14, 15] in primary care settings have been developed and well tested in Western nations. Based on the chronic disease management model [16], they typically include the use of algorithm-driven, evidence-based depression treatments administered by the PCP in collaboration with a depression-care specialist (nurse, psychologist, social worker) who is embedded in the practice and supervised by a psychiatrist; regular symptom monitoring; and education and support of patients and families to ensure their active engagement in care. This integrated or collaborative depression care management approach has been associated with improved clinical outcomes as well as cost savings relative to care-as-usual (CAU), including in older adults [14, 17]. It has rarely been applied in developing nations like China, however. One exception is a randomized controlled trial of collaborative depression care management among older adults in urban primary care clinics conducted by our group, the results of which showed significantly greater reductions of depressive symptoms over 12 months when compared with patients who received CAU [18]. No RCTs have examined the effectiveness of integrated depression care management in rural China, where access to care is more challenged, none has addressed depression comorbid with other common chronic conditions such as HTN, and none has complemented medication management with a psychosocial treatment component, which is often a preferred option of older adult Chinese [19].

Our objective with this study is to address these shortcomings of previous work. Using a cluster randomized controlled design, the Chinese Older Adult Collaborations in Health (COACH) Study will compare the COACH intervention to enhanced Care-as-Usual (eCAU) for the treatment of comorbid depression and HTN in older residents of rural Chinese villages. COACH integrates the care provided by a PCP with that delivered by an Aging Worker (AW, a staff person of either the Women’s Federation or Aging Association of the village), and supervised by a psychiatrist consultant (Psychiatrist). The study’s five aims are designed to test the hypotheses that, relative to subjects who receive eCAU, those in the COACH intervention will show (Aim 1a) better adherence to antidepressant treatment recommendations and (Aim 1b) greater improvements in their depressive disorders over 12 months of involvement in the study; and further that relative to subjects who receive eCAU, those in the COACH intervention will show (Aim 2a) better adherence to antihypertensive treatment recommendations and (Aim 2b) greater improvements in blood pressure (BP) control. We will examine the temporal associations of change in depression and BP control, hypothesizing that (Aim 3a) improvements in treatment adherence precede symptomatic improvement in depression and hypertension, (Aim 3b) improvements in depression will precede improvements in BP control, and (Aim 3c) those temporal patterns are associated with the intervention received. We hypothesize also that the COACH intervention results in greater improvements in health-related quality of life than eCAU (Aim 4). Finally (Aim 5), we will explore resource utilization and costs associated with delivering the eCAU and the COACH interventions over 12 months, important information with which to guide subsequent research on implementation and dissemination of the model if it is shown to be clinically effective.

## Methods

### Setting

This ongoing study is being conducted in rural villages of Tonglu and Jiande counties, two of 55 counties in Zhejiang Province, China. With population size and average household income at the mean level of all counties in Zhejiang Province, Tonglu and Jiande encompass 283 and 319 villages respectively, with average populations of 1400 and 1600. Approximately 20% of their populations are age ≥ 60 years. They are served by a total of 602 PCPs (one in each village) and two mental hospitals (one for each county), which combined have 19 psychiatrists who serve 0.72 million village residents. Every village has either a Women’s Federation or an Aging Association that is staffed by local residents, one of whom is the designated AW.

### Sample strategy and procedures

As a guard against bias associated with “leakage” of the intervention across arms of the study, the unit of randomization is the village, which is equivalent to clinic-level randomization. Of all villages in Tonglu and Jiande County 160 have been randomly selected by Prof. Hengjin Dong to receive either the eCAU or COACH. No two villages assigned to different intervention arms (COACH or eCAU) are geographically contiguous. Upon random selection of each village, each PCP and AW are approached by the study coordinator and their county-level supervisors for agreement to participate. If any PCP or AW declines, or if their assignment places them immediately adjacent to a village in which the other intervention is to be offered, another village will be selected from the pool.

### Subjects

A total of approximately 2400 subjects will be recruited to participate in the study for up to 12 months. They will include registered residents who live independently in the selected villages in Tonglu or Jiande County. They must be ≥60 years and have both clinically significant depression, defined as a baseline score on the Patient Health Questionnaire-9 (PHQ-9) [20] of 10 or more, and a diagnosis of HTN in their medical record. Subjects must have intact cognitive functioning (Six-Item Screener [SIS] [21] score < 3) and be capable of participating in study interviews with informed consent. Subjects found on baseline assessment to have mania, psychosis, alcohol abuse or dependence active in past 6 months, or suicidal ideation with intent are excluded from the study with a recommendation made to the PCP that they be referred for psychiatric assessment at the County Mental Hospital.

### Procedure for recruitment, consent and retention

Each village clinic has a standardized electronic medical record system (EMR) supported by the Zhejiang Center for Disease Control and Prevention (CDC) in which each patient is registered. PCPs use the EMR to identify all village residents age ≥ 60 years who have a diagnosis of HTN. Trained in the administration of the PHQ-9, they then visit each potential subject, either in their home or the village clinic, and administer the PHQ-9. Those who screen positive are invited to meet with the research assistant (RA) who introduces him or her to the study, obtains written informed consent, and conducts the baseline research assessment. RAs return to the village 3, 6, 9, and 12 months later to conduct follow up research interviews. The subjects’ involvement in the study ends after 12 months.

### Comparison condition: Enhanced Care-as-Usual (eCAU)

PCPs in village clinics have on average three years of medical education after completing high school. They receive systematic training in the diagnosis and treatment of common chronic medical illnesses. For HTN management they are expected to follow guidelines developed by the Center for Disease Control and Prevention in China, [21] which is analogous to those developed by the U.S. Joint National Committee on Prevention, Detection, Evaluation, and Treatment of High Blood Pressure (the “JNC 7 Report” [22]). The guidelines address detection, management by both pharmacologic and non-pharmacologic means, and guidance on when to refer to more specialized care.

Village PCPs, however, receive little training in the diagnosis or treatment of mental disorders and ordinarily are provided with no practice guidelines to help with its management. When depression is suspected by PCPs, current practice involves suggesting to patients (or family members) that they consult a psychiatrist at the County Mental Hospital for diagnosis and treatment. There is no direct referral/transfer mechanism between PCPs and mental health specialists, and it is uncommon for patients to take the initiative. Provincial laws prohibit the village PCP from initiating antidepressant treatment. However, if the patient does see the County Mental Hospital psychiatrist who then begins antidepressant medication, the PCP may choose to renew the prescription, typically in two-week increments.

We refer to CAU as “enhanced” (eCAU) because PCPs are told when study subjects screen positive for depression and are provided with copies of depression practice guidelines developed for the study. Because of constraints on their knowledge and practice, however, we do not anticipate that access to this information will substantially impact patient outcomes [23–27].

### Intervention: COACH

The COACH intervention applies chronic disease management principles to the treatment of both depression and HTN by a team consisting of the village PCP, AW, and a Psychiatrist.

#### Primary care providers (PCP)

The background and qualifications of the PCP are as described above for eCAU. The PCP’s role and responsibilities in COACH include screening and ongoing monitoring of blood pressure and depressive symptoms with the PHQ-9, management of depression and HTN using standardized practice guidelines in collaboration with other COACH team members, and education of the patient, family and community about these chronic conditions.

#### Aging Workers (AW)

A staff member of either the village’s Women Federation or Aging Association is designated as the AW for the COACH team. S/he typically has a middle-school education, knows the residents well, and is well connected with the village leadership. Although the AWs have no special training in social work, they receive in-service training from the Bureau of Civil Affairs and have experience in addressing the villagers’ social needs. Their responsibilities for the COACH intervention include baseline and ongoing assessment of social stressors and supports, fostering social connectedness, behavioral activation, adherence support, facilitating communications with the PCP, and education of the patient, family and community about depression and HTN and their management.

#### Psychiatrist

Each COACH team is linked with one of the psychiatrists based at the Tonglu and Jiande County Mental Hospital. These two hospitals have a full array of inpatient (40 and 42 beds respectively) and outpatient services. Psychiatrists have on average 5 years of medical training after high school followed by 2 years of specialty training. Their scope of practice includes psychiatric diagnosis and management of patients of all ages with mental illness from across the county. Their role in COACH is to provide baseline diagnosis and initial prescription of depression treatment, and to provide ongoing consultation to the team in its algorithm-driven management of the patient’s illness.

### Training requirements and procedures

Following randomization, PCPs and AWs of villages assigned to implement COACH undergo training at the County Mental Hospital along with the Psychiatrist consultant. Each member of the COACH team requires training in their individual role, and all require training in how to work together collaboratively.

The intervention as outlined in the curriculum for the PCPs includes four major components: (a) depression management guidelines adapted from the Duke Somatic Treatment Algorithm for Geriatric Depression (STAGED) [28] and applied in our prior study of depression care management in urban Hangzhou clinics [18]; (b) hypertension management using the guidelines adopted by the primary care system in China [21]; (c) case management using the toolkit adapted from the MacArthur Initiative on Depression in Primary Care [21, 29]; and (d) psychoeducation (PE) and communication skills. PCP training is administered over 4 days. There are no restrictions on concomitant care or treatment,

The AW curriculum, administered over 3 days, includes: (a) an overview of depression, HTN, and their relationship; (b) self-management of depression and hypertension; (c) social environment as risk and protective factors; (d) conducting psychosocial assessment and developing a care plan; (e) providing psychoeducation to subjects and their families; (f) techniques to reinforce treatment adherence and healthy lifestyles; (g) communication and problem-solving skills with older adults and their families; (e) ethical standards including confidentiality.

Finally, PCPs, AWs and Psychiatrists come together for 1 day to learn how to collaborate in care management. They are taught about the roles of the other team members and review procedures guiding their communications and information transfer. Instruction techniques include structured didactics, role-plays, and process/problem solving sessions exploring potential barriers to communication and collaboration.

PCPs, AWs, and Psychiatrists in the COACH study arm and PCPs in eCAU villages receive a small salary stipend from the Departments of Health of Tonglu and Jiande counties for their added effort in support of the study.

### Procedures guiding team implementation of COACH

With initiation of the study the PCP meets with each subject to review management of their BP. The Psychiatrist travels to the village and conducts the assessment to establish the depression diagnosis in the subject’s home or the village clinic according to the subject’s preference. S/he then consults with the PCP and, if medications are indicated, initiates treatment with an antidepressant according to the study’s treatment guidelines. The AW visits the subject in his or her home to conduct a systematic assessment of the person’s functional status, social supports, lifestyle, medication use, nutritional and financial status. The AW and PCP then meet, review the findings of the AWs in-home assessment and the patient’s physical and mental health, and construct a care plan (problem list, approach to each problem, responsible person, target date for completion) that addresses social, physical, and mental health needs in a coordinated fashion.

The PCP continues to meet with the patient at the intervals designated by the HTN and depression practice guidelines, monitoring progress with repeated PHQ-9 and BP measures and adjusting treatment as indicated. The AW continues to work with the subject to address identified problems; educate the subject and family about their illnesses and support adherence to depression and hypertension treatment regimens; encourage good health behaviors (diet, exercise, smoking cessation) based on established psychoeducation methods; and connect the patient to others and to the community (e.g., engage in social groups, visits with friends and family). These activities can be through working with individuals (e.g., home visits with the subject and family), small groups (e.g., cooking demonstrations, Tai Chi class), and community events (e.g., communal meals, games.) AW outreach to the subject continues weekly for 2 months, then biweekly for 2 months, then monthly. If the patient continues to require AW support, more frequent visits are conducted.

The PCP and AW are instructed to meet weekly to review their shared caseload. Each patient is very briefly discussed and the treatment plan updated as indicated. The Psychiatrist is available by telephone to make additional suggestions regarding depression management, and may refer the patient to the County Mental Hospital as needed to assure safety.

### Study measures and procedures

Study measures are administered in face-to-face interviews conducted in the subject’s home or the clinic by unblinded, trained research assistants at study entry and 3-, 6-, 9-, and 12-months later. Data will be transferred from hard copy forms by duplicate entry to the electronic data base and maintained in secure servers at the Zhejiang University. Reasons for dropout and any occurence that could constitute an adverse event will be documented by the research interviewers, examined by the study investigators, and reported as indicated to the study’s institutional review boards. As well, they will be assessed in semi-annual meetings of the study’s Data and Safety Monitoring Board (DSMB) that is composed of Chinese experts independent from the sponsor and competing interests.

The measures we obtain fall into three domains: socio-demographic and baseline characteristics, outcome variables, and covariates that may independently influence adherence and treatment response. The timing of their administration is depicted in Table 1.

**Table 1:** Timeline of measures

Measure	AIM	Baseline	1 months	3 months	6 months	9 months	12 months
HDRS, PHQ-9		x	x	x	x	x	x
BP		x	x	x	x	x	x
MPR		x					x
WHOQOL-BREF		x			x		x
BMI		x					x
CCI		x					x
ADL, IADL		x			x		x
MOS-SSS-C, SNS		x			x		x
CSQ-8		x					x
PS		x					x
Cost data (EMR)		x					x

Notes: *ADL* activities of daily living, *BMI* body mass index, *BP* blood pressure, *CCI* Charlson Comorbidity Index, *CSQ-8* Client Satisfaction Questionnaire 8-item, *EMR* electronic medical record, *HDRS* Hamilton Depression Rating Scale, *IADL* instrumental activities of daily living, *MOS-SSS-C*, Medical Outcomes Study Social Support Survey, *MPR* medication possession ratio, *SNS* Social Network Size, *PS* Perceived Stigma Scale, *WHOQOL-BREF* WHO Quality of Life-BREF

Socio-demographic characteristics include age, sex, education and literacy level, and marital status. During the baseline interview we use the Mini-International Neuropsychiatric Interview (MINI) [30] to assess for the presence in the last 6 months of mania, psychosis, and alcohol misuse, which are exclusion criteria. If a question of suicide risk is raised, the study’s safety protocol is implemented and a decision made whether the subject should be excluded from the study.

Our primary outcome variables are adherence to antidepressant and antihypertensive medication recommendations, depression symptom severity, and BP control. Adherence is measured both by self-report (a single question inquiring whether the subject has missed any doses of her/his medications in the past) and by construction of a Medication Possession Ratio (MPR) from prescriptions written and filled, based on the patient’s EMR. The MPR then is calculated as the ratio of the total days of medication supplied to total days in a period [31], with values ≥80% considered medication adherent [32]. The MPR is an accepted metric for the evaluation of adherence using retrospective data, including in China [33].

The measure of depressive symptom change will be the Hamilton Depression Rating Scale – 17 item version (HDRS), which is widely used, reliable and valid in Chinese [34]. Consistent with other geriatric depression studies [35] and our own work [36], we define response to treatment as a change in the HDRS score > 50% from baseline, and remission as a follow-up HDRS score lower than 7.

BP control is established at follow up using guidelines specified by the Hypertension Treatment Guidelines [21, 22]. BP is taken according to the standards developed by the Center for Disease Control and Prevention in China (auscultation with cuff deflation method after seated quietly for 5 min in the proper position; no caffeine, exercise or smoking in preceding 30 min; appropriately sized cuff; average of three measurements is recorded.) For analyses, uncontrolled HTN is defined as systolic BP ≥ 130 or diastolic BP ≥ 80 for patients with diabetes mellitus, coronary heart disease or renal disease, and BP ≥ 140 or diastolic BP ≥ 90 for all others [21, 22].

Quality of life is measured using the WHOQOL-BREF [37, 38], a 26 item scale that yields four domain scores - physical, psychological, social and environment - each of which we will examine separately in analyses. The WHOQOL-BREF is a widely used measure the Chinese version of which has shown very good psychometric properties [39–41].

In exploratory analyses we will estimate the costs associated with two components of the intervention: (a) the incremental costs of adding COACH resources to eCAU (programmatic costs), and (b) medical costs attributable to the care of the subjects in each arm. Programmatic costs will include expenses associated with training, travel for consultant psychiatrists between Tonglu/Jiande and the villages for the intake assessment, staff time for intervention team meetings, and any information system costs. Medical costs include costs of medical treatment documented by the subjects’ EMR and Zhejiang Province insurance data that document each office visit, drug prescription, laboratory test performed, or hospital stay for medical or mental health reasons. As well, we will include patient out-of-pocket healthcare costs in the preceding 3-month interval obtained by subject interview at each research assessment point. Cost data will be converted to US dollars.

Additional variables for use as covariates in analyses include factors known to influence adherence, treatment response or both, include the Charlson Comorbidity Index [42] adapted for use with ICD-9 codes [43] and supplemented by questions about common disabling conditions of late life including diabetes, high cholesterol, heart disease, and stroke; and the Body Mass Index (BMI), which is associated with hypertension treatment response. As a measure of functioning independent of the WHOQOL-BREF physical domain score, we measure the subject’s impairment in basic (ADL) and instrumental activities of daily living (IADLs) [44] and cognitive function with the Six-Item Screener (SIS) [45, 46]. We measure social support with the Chinese version of the Medical Outcomes Study Social Support Survey (MOS-SSS-C) [47] and social network size (total number of persons with whom the respondent has discussed important matters in the past 6 months) [48]. Participants will be queried about side effects of both their antidepressant and antihypertensive medications using the Antidepressant Side-Effect Checklist [49] and the Side-Effects and Symptoms Distress Checklist [50] respectively.

### Sample size estimate

Primary outcomes of the COACH Study on which sample size estimates were based are (1) adherence to depression and HTN treatment (MPR and % of subjects with 80% MPR); (2) depression symptom change (proportion with 50% reduction in HDRS and % with HDRS < 10); and (3) HTN control (% with BP controlled). Additional assumptions on which selection of the estimated sample of 2400 from villages randomized by village to the COACH or eCAU conditions was based include an attrition rate of 20% over 12 months, informed by our previous studies in Hangzhou. Since the village-randomized study is a 3-level nested longitudinal design, power depends on the intra-class correlation (ICC) among the patients within the PCP and serial correlation between repeated assessments within the patient. We set the serial correlation at 0.5, but varied the ICC over 0.05, 0.1 and 0.2 to get a sense of the impact of the latter on power. Based on two-sided type I error = 0.05, power = 0.8 and an attrition rate of 20%, the detectable effect size ranged from 0.17 to 0.26 for a continuous outcome. Since experiences with multi-level designs suggest that ICC is generally smaller than 0.2, the study is sufficiently powered to detect small effect sizes between the two intervention groups. For the dichotomous outcomes, we also set the serial correlation at 0.5 and varied the percent of variance between patients within the PCP over 0.05, 0.1 and 0.2. Based on two-sided type I error = 0.05, power = 0.8, base rate 0.5 (most conservative) and an attrition rate of 20%, the detectable between-group proportion ranged from 11 to 17%, which is well within the range of clinically meaningful differences in the primary care setting.

### Statistical analytic approach

Descriptive statistics (counts and proportions for categorical variables and means [± SD] for continuous outcomes) will be used to depict the characteristics of the sample (e.g., age, gender, physical and functional status). We will compare baseline characteristics between groups using t-test (for continuous variables) and chi-square (for discrete variables), and examine associations between outcome variables and patients’ characteristics. Characteristics significantly differentiating the two groups will be treated as covariates when testing between-group differences using longitudinal models and structural equation models (SEM).

#### Aim 1 – Depression

We hypothesize that relative to subjects who receive eCAU, those in the COACH intervention will show (a) better adherence to antidepressant treatment recommendations and (b) greater improvements in their depressive disorders over 12 months of involvement in the study. We will model each repeatedly assessed variable of adherence to depression treatment as well as improvement in depression using generalized linear mixed effect models (GLMM) and weighted generalized estimating equations (WGEE), two common approaches for modeling treatment differences over time [51]. Both approaches provide valid inference in the presence of missing data if the missing value follows the missing at random (MAR) assumption, a popular mechanism that applies to most studies in practice [51]. If estimates differ between the two approaches, only WGEE results will be reported, as it provides significantly more robust inference, especially in the presence of missing data [51, 52]. We will perform intention-to-treat analyses using all subjects randomized to the treatment groups.

For each outcome, time and intervention will be predictors, adjusting for covariates. We will also assess the potential interaction between time and intervention. To avoid oversimplifying temporal patterns with linear trend, we will use piece-wise or even polynomial functions of time based on the assessment points. Linear contrasts will be used to assess COACH vs. eCAU differences over the 12-month period as well as any sub-intervals within this period.

#### Aim 2 – Hypertension

We hypothesize that relative to subjects who receive eCAU, those in the COACH intervention will show (a) better adherence to antihypertensive treatment recommendations and (b) greater improvements in BP control. The same approach as Aim 1 will be used to examine the hypotheses in this Aim.

#### Aim 3 – Temporal associations

We will examine the temporal associations of change in depression and BP control, hypothesizing that (a) improvements in treatment adherence precede improvement in depression and hypertension, and (b) improvements in depression will precede improvements in BP control. The dynamic relationships between adherences, improvement in depression and BP control and mediation of the intervention effect on BP control by improvement in depression will be examined using SEM. We will first examine the causal, or meditational, relationship between the COACH intervention, adherence to antidepressant (antihypertensive) medications and improvement in depression (BP control), using SEM with intervention as the predictor, adherence as the mediator and depression (BP control) as the outcome. To ensure the temporal order that changes in adherence take place before improvements in depression (BP control), the depression (BP control) variable will lag by one visit with respect to the adherence variable. We will construct direct, indirect, and total effects, and perform tests to see if adherence to antidepressant (antihypertensive) medications mediates the effect of the intervention on improvement in depression (BP control).

The same approach will be used to test the mediation hypothesis in (b), where depression is the mediator and BP control is the outcome.

#### Aim 4 – Health-related quality of life

In addition to its impact on depression and BP control, we will ask whether the COACH intervention results in greater improvements in health-related quality of life than eCAU. We will use the same approach as in Aim 1 to test the hypothesis. The longitudinal model will be applied to each of the WHOQOL-BREF domain scores.

#### Aim 5 – Cost

Finally, we will determine resource utilization and costs associated with delivering the eCAU and the COACH interventions over 12 months and, in a set of exploratory analyses, conduct an economic feasibility evaluation using cost-effectiveness analysis (CEA) and cost-benefit analysis (CBA) methods. We consider them exploratory because the 12-month duration of the intervention is relatively short for such analyses.

For CEA we will calculate an incremental cost-effectiveness ratio (ICER) based on two outcomes: (a) reduction in the average HDRS score between the eCAU and COACH subjects (ΔEffect = hypertension [eCAU] – hypertension [Coach]); and (b) Increase in the proportion of patients with controlled BP: ΔEffect = cBP(eCAU) – cBP(Coach). The ICER will be interpreted as the incremental cost per unit improvement in health as measured by the outcome of choice (HDRS score or % of patients with BP control).

For CBA, the benefit of the intervention will be estimated as the reduction in the average healthcare expenses between the patients receiving eCAU and COACH (ΔEffect = ΔBenefit = MedCost[CAU] – MedCost[Coach]). The results of cost-benefit analysis could be interpreted as the return on investment (ROI; the ratio of reduction in medical costs to the incremental cost of the intervention, with ratio > 1 indicating a significant return) or as a net monetary benefit (NMB = the difference between the reduction in medical costs and the incremental cost of the intervention, NMB > 0 indicating a monetary benefit of the intervention).

## Discussion

The COACH study uses a cluster randomized controlled trial design to compare an integrated, primary care-based depression care management approach to eCAU for the treatment of comorbid depression and HTN in older adult Chinese rural village residents. Building on a large body of research that has established the effectiveness of integrated care models for depression and other common mental disorders in Western countries, the COACH study is one of very few to apply the model in a low/middle income country (LMIC) in which mental health resources are scarce and the need is enormous and growing rapidly. With 150 million adults over age 65 currently, China’s older adult population is projected to grow to over 400 billion by 2050, even as the relative proportion of the working aged population decreases. Rapid changes are underway in family structure with migration of younger and middle aged adults from rural to urban centers for work, leaving their older adult parents behind in rural villages where the prevalence of depression is high and treatment is rarely accessible. Indeed, this situation is common in the developing world. Research is required on means by which to give primary care clinics the tools with which to address the mental health needs of the aged population in rural areas efficiently and effectively.

Although our previous study of depression care management in urban Chinese primary care clinics produced promising results for treatment of late life depression, it was limited in a number of respects that the COACH study will attempt to address. COACH is implemented in rural villages, a necessary innovation given that 70% of the older population lives in rural China. As well, COACH incorporates a psychosocial dimension to care with the AW that was lacking in previous work, couples depression with care management of HTN to test the model’s impacts on commonly comorbid chronic conditions, and will contribute to understanding the role that adherence plays in chronic disease management.

Successful dissemination of the COACH model to other counties and provinces in China, and to other rural LMIC settings, will require that it is more effective than eCAU in reducing depressive symptoms, improving quality of life, and improving BP control. It will require that the delivery model be acceptable to multiple stakeholders in diverse sociocultural contexts, including the patients and their families, PCPs and other team members, village leaders and those who administer the health and aging services systems. To that end, COACH is designed to leverage indigenous village resources to contain costs and to meet the needs of village residents and providers in a manner that is culturally congruent.

Successful dissemination will also require that the model be affordable, for which this study will provide some preliminary evidence. Nonetheless, additional modifications will likely be needed, for example, by linking the COACH teams to psychiatric supports using telehealth technology, and telementoring using the Project ECHO^®^ model to build the teams’ expertise over time, reducing the need for ongoing support and improving cost efficiency. With these additional steps, Zhejiang Provincial officials may support further testing on a larger scale.

## Abbreviations

ADL: Activities of daily living
AW: Aging worker
BMI: Body mass index
BP: Blood pressure
CBA: Cost-benefit analysis
CDC: Center for disease control and prevention
CEA: Cost-effectiveness analysis
COACH: Chinese Older Adult Collaborations in Health
CSQ-8: Client satisfaction questionnaire 8-item
CVD: Cardiovascular disease
DM: Diabetes mellitus
eCAU: Enhanced Care-as-Usual
EMR: Electronic medical record
HDRS: Hamilton depression rating scale
IADL: Instrumental activities of daily living
ICER: Incremental cost-effectiveness ratio
LMICs: Low-and middle-income countries
MINI: Mini International Neuropsychiatric Interview
MOS-SSS-C: Medical outcomes study social support survey
MPR: Medication possession ratio
PCP: Primary care provider
PHQ-9: Patient Health Questionnaire-9
RA: Research assistant
RCT: Randomized controlled trial
SEM: Structural equation models
SIS: Six-item screener
STAGED: Somatic treatment algorithm for geriatric depression
VAA: Village aging associations
WHOQOL-BREF: WHO Quality of Life-BREF
